# African Swine Fever Virus: An Emerging DNA Arbovirus

**DOI:** 10.3389/fvets.2020.00215

**Published:** 2020-05-13

**Authors:** Natasha N. Gaudreault, Daniel W. Madden, William C. Wilson, Jessie D. Trujillo, Juergen A. Richt

**Affiliations:** ^1^Department of Diagnostic Medicine/Pathobiology, College of Veterinary Medicine, Kansas State University, Manhattan, KS, United States; ^2^Arthropod Borne Animal Diseases Research Unit, Agricultural Research Service, United States Department of Agriculture, Manhattan, KS, United States

**Keywords:** African swine fever virus, DNA arbovirus, soft tick, domestic swine, wild boar, transmission, virus replication

## Abstract

African swine fever virus (ASFV) is the sole member of the family *Asfarviridae*, and the only known DNA arbovirus. Since its identification in Kenya in 1921, ASFV has remained endemic in Africa, maintained in a sylvatic cycle between *Ornithodoros* soft ticks and warthogs (*Phacochoerus africanus*) which do not develop clinical disease with ASFV infection. However, ASFV causes a devastating and economically significant disease of domestic (*Sus scrofa domesticus)* and feral (*Sus scrofa ferus*) swine. There is no ASFV vaccine available, and current control measures consist of strict animal quarantine and culling procedures. The virus is highly stable and easily spreads by infected swine, contaminated pork products and fomites, or via transmission by the *Ornithodoros* vector. Competent *Ornithodoros* argasid soft tick vectors are known to exist not only in Africa, but also in parts of Europe and the Americas. Once ASFV is established in the argasid soft tick vector, eradication can be difficult due to the long lifespan of *Ornithodoros* ticks and their proclivity to inhabit the burrows of warthogs or pens and shelters of domestic pigs. Establishment of endemic ASFV infections in wild boar populations further complicates the control of ASF. Between the late 1950s and early 1980s, ASFV emerged in Europe, Russia and South America, but was mostly eradicated by the mid-1990s. In 2007, a highly virulent genotype II ASFV strain emerged in the Caucasus region and subsequently spread into the Russian Federation and Europe, where it has continued to circulate and spread. Most recently, ASFV emerged in China and has now spread to several neighboring countries in Southeast Asia. The high morbidity and mortality associated with ASFV, the lack of an efficacious vaccine, and the complex makeup of the ASFV virion and genome as well as its lifecycle, make this pathogen a serious threat to the global swine industry and national economies. Topics covered by this review include factors important for ASFV infection, replication, maintenance, and transmission, with attention to the role of the argasid tick vector and the sylvatic transmission cycle, current and future control strategies for ASF, and knowledge gaps regarding the virus itself, its vector and host species.

## ASFV Emergence and Re-emergence

Since its identification in Kenya in 1921 (1, 2), African swine fever virus (ASFV) has remained endemic in Africa, affecting up to 35 African countries (3). Between the late 1950s and early 1980s, ASFV genotype I emerged in Europe, Russia, the Caribbean and South America. ASFV was first identified in Europe in 1957 in Portugal, then was re-introduced in 1960 from which it quickly spread into Spain, Italy, France, Sardinia, Malta, Belgium, and The Netherlands (1, 4, 5). ASFV was first reported in Russia in 1977 (4), and in the late 1970s it emerged in Brazil, Cuba and the Caribbean Islands, with the last outbreaks in the Americas occurring between 1980 and 1984 (6). By the mid-1990s, ASFV had been eradicated outside of Africa, with the exceptions of an isolated outbreak in Portugal in 1999 and the island of Sardinia where it has remained endemic (7, 8).

**Figure 1:**
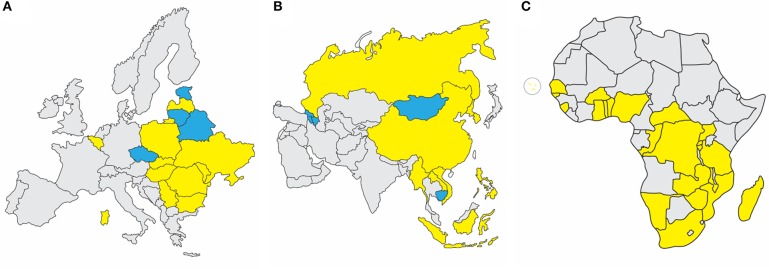
Recent ASF status in Europe, Asia, and Africa. **(A)** Eurasian Epidemic, 2007-September 2019: Within European nations, continuing outbreaks (yellow) are reported in Sardinia, Belgium, Bulgaria, Hungary, Latvia, Moldova, Poland, Romania, Slovakia, Serbia, Russian Federation, and Ukraine. Resolved outbreaks (blue) are reported for Belarus, Czech Republic, Estonia, and Lithuania. **(B)** Transcaucasus and Asian Epidemic, 2007-September 2019: Continuing outbreaks (yellow) are reported in People's Republic of China, Democratic People's Republic of Korea, Lao People's Democratic Republic, Myanmar, The Philippines, Russian Federation, Republic of Korea, and Vietnam. Resolved outbreaks (blue) include Armenia, Azerbaijan, Cambodia, Republic of Georgia, and Mongolia. **(C)** African Nations with OIE-Notified ASF Outbreaks Since 2018: Countries which have notified the OIE of the presence of ASF from 2018 through September 2019 include Benin, Burkina Faso, Burundi, Cabo Verde, Central African Republic, Democratic Republic of the Congo, Gambia, Ghana, Guinea-Bissau, Madagascar, Malawi, Mozambique, Namibia, Nigeria, Rwanda, Senegal, Sierra Leone, South Africa, Tanzania, Togo, Uganda, Zambia, and Zimbabwe. Source: OIE WAHIS African Swine Fever (ASF) Report: September 13–26, 2019.

In 2007, ASFV genotype II emerged in the Republic of Georgia and continued to spread through the Caucasus region and subsequently into the Russian Federation and Eastern Europe, where it has continued to circulate and spread as illustrated in Figure 1A (1, 4, 9). ASFV re-emerged in north-western Europe in Belgium in 2018 in wild boar (10). More recently it was detected in carcasses of wild boar in western Poland near the German boarder (https://www.vettimes.co.uk). In August 2018, ASFV was reported for the first time in the People's Republic of China, and by the end of September of 2019, ASFV was detected in neighboring countries including Mongolia, Vietnam, Cambodia, Democratic People's Republic of Korea (North Korea), Lao People's Democratic Republic, Myanmar, Timor-Leste, the Philippines, the Republic of Korea (South Korea), and Indonesia as shown in Figure 1B (FAO situation update, www.fao.org). African countries which have notified the OIE of the presence of ASF from 2018 through September 2019 include Benin, Burkina Faso, Burundi, Cabo Verde, Central African Republic, Democratic Republic of the Congo, Republic of the Congo, The Gambia, Ghana, Guinea-Bissau, Madagascar, Malawi, Mozambique, Namibia, Nigeria, Rwanda, Senegal, Sierra Leone, South Africa, Tanzania, Togo, Uganda, Zambia, and Zimbabwe, indicated in Figure 1C [OIE WAHIS African Swine Fever (ASF) Report: September 13–26, 2019; www.oie.int].

### Impact of Recent ASFV Emergence as of February 2020

ASFV does not cause disease in humans, is highly contagious and causes high mortality in domestic and feral swine, and has a significant economic impact on the global swine industry. While the situation remains ever-changing due to continued outbreaks and spread of ASFV globally, information from peer-reviewed manuscripts, situation reports, and press releases provide some indication of the impact of ASFV emergence on animal health and economics of effected countries. Table 1 summarizes the ASF affected countries of Europe and Asia from 2007 to February 2020 including reported estimates of number of animals lost. ASF has especially affected China, which is the world's largest pork producer and consumer, producing about 50 percent of the world's pork supply (ChinaDaily.com.cn, 9/11/2019, “Swine fever may affect pork for several years,” global.chinadaily.com.cn). Since the first reported outbreak in China in August 2018, ASF has been detected in at least 8 other countries in Asia and has resulted in the death or culling of more than 5 million pigs, with losses accounting for more than 10 percent of the total pig population in China, Mongolia and Vietnam [(11, 12); FAO situation update, www.fao.org; FAO press release, 09/08/2019, “One year on, close to 5 million pigs lost to Asia's swine fever outbreak”], and industry insiders predict a 30–60% loss of pig stocks due to ASF (ChinaDaily.com.cn, 9/11/2019, “Swine fever may affect pork for several years,” global.chinadaily.com.cn). This has put other countries on high alert, including Thailand which culled 200 pigs in response to mysterious pig deaths although no confirmed cases of ASF had been reported, as of September 2019 (Reuters Health News, 09/18/2019, “Thailand culls 200 pigs amid heightened fears over African swine fever,” www.reuters.com). Since its identification in 1921, outbreaks of ASFV have been reported in more than 60 countries around the world, and global ASF outbreaks since late 2018 have increased 25 percent according to media reports (Global Times, 09/18/2019, “A global battle against African swine fever,” www.globaltimes.cn).

**Table 1:** ASFV in Eurasia from January 2007 to February 2020.

**Country**	**Year or date reported**	**Status**	**Estimated animal losses**	**Species**
Georgia	2007–2008	Resolved	87,412	Swine
Armenia	2007–2008, 2010–2011	Resolved	2,483	Swine
Azerbaijan	2008	Resolved	4,832	Swine
Russian Federation	2007–2019	Continuing	79,632	Swine, wild boar
Ukraine	2012, 2014–2019	Continuing	20,166	Swine, wild boar
Belarus	2013	Resolved	20,627	Swine
Lithuania	2014–2019	Resolved	23,735	Swine
Latvia	2014–2019	Continuing	294	Swine, wild boar
Estonia	2014–2019	Resolved	26	Wild boar
Poland	2014–2019	Continuing	37,396	Swine, wild boar
Czech Republic	2017, 2018	Resolved	202	Wild boar
Romania	2017–2019	Continuing	90,698	Swine, wild boar
Hungary	2018–2019	Continuing	1,536	Wild boar
Bulgaria	2018–2019	Continuing	137,973	Swine, wild boar
Moldova	2016–2019	Continuing	348	Swine, wild boar
Belgium	2018–2019	Continuing	540	Wild boar
Slovakia	2019	Continuing	70	Swine, wild boar
Serbia	2019	Continuing	290	Swine
People's Republic of China/32 provinces	August 3, 2018	Continuing	1,193,000	Swine, wild boar
Mongolia/6 provinces	January 15, 2019	Resolved	3,115	Swine
Vietnam/19 provinces	February 19, 2019	Continuing	5,960,000	Swine
Cambodia/5 provinces	April 2, 2019	Resolved	3,673	Swine
Democratic People's Republic of Korea	May 23, 2019	Continuing	124	Swine, wild boar
Lao People's Democratic Republic /15 provinces	June 20, 2019	Continuing	40,130	Swine
The Philippines	July 25, 2019	Continuing	70,000	Swine
Myanmar	August 1, 2019	Continuing	128	Swine
Republic of Korea	September 17, 2019	Continuing	10,000	Swine, wild boar
Timor-Leste	September 9, 2019	Continuing	1,600	Swine
Indonesia	September 2019	Continuing	42,000	Swine

*FAO situation update, www.fao.org, 02/19/2020; OIE WAHIS Interface, Disease information, Immediate notifications and Follow-ups, www.oie.int, 09/21/2019; OIE WAHIS Interface, Disease information, Disease Timelines, www.oie.int, 10/23/2019*.

## ASFV Infection, Maintenance, and Transmission

### *Ornithodoros* Soft Ticks

The *Ornithodoros* genus of soft ticks in the family *Argasidae* serve as biological vectors and reservoir hosts for ASFV. To date, eight *Ornithodoros* species have been demonstrated as vector competent for ASFV (13). ASFV-infected *Ornithodoros porcinus porcinus* soft ticks (often referred to as *O. moubata porcinus* or *O. moubata*) in Africa have been well-documented (14–19) and have also been found in Madagascar (20). Additionally, competent *Ornithodoros* vectors are also known to exist in parts of Europe and the Americas (13, 18). *Ornithodoros erraticus* (also known as *O. marocanus* and renamed *Carios erraticus*) soft ticks inhabit the Iberian Peninsula and Mediterranean areas of Africa and Asia, and were an important vector and reservoir for ASFV in Portugal and Spain during the ASF epidemic in the twentieth century (7, 21–23).

*Ornithodoros* ticks have long lifespans, and ASFV can replicate to high titers and be maintained for long periods of time in the vector with minimal cytopathological effects or increased tick mortality (7, 14–18, 20, 24, 25); although increased mortality rates have also been reported (26–31). A study following ASFV infection in *O. porcinus porcinus* ticks after feeding on viremic pigs showed ASFV titers of 6 log_10_ HAD_50_/tick, which were maintained at that level for at least 290 days and declined only 2 log_10_ HAD_50_/tick or less after 3 years (18, 25). ASFV was isolated from *O. moubata* ticks from a farm in Madagascar 4 years after the culling of all pigs (20). ASFV transmission to pigs by infected the Iberian soft tick has been demonstrated up to 588 days after infection (29) and ASFV persistence has been shown for at least 5 years in *O. erraticus* ticks collected from infected farms in Portugal (7). However, viral clearance after one year has also been observed (28, 32). Nonetheless, virus-tick adaptation is likely necessary to achieve high virus titers since significantly lower infection rates and viral titers, and increased mortality have been observed in studies using ASFV isolates not derived from ticks, or *Ornithodoros* species not native to Africa (18, 25, 33).

Multiple ASFV genetic elements have been identified as being associated with infectivity, replication, and generalized dissemination of ASFV in *Ornithodoros* ticks. Deletion of three multigene family (MGF) 360 genes (*3Hl, 3IL*, and *3LL*) from the tick-derived pathogenic ASFV Pr4 isolate resulted in reduced infectivity and a 2–3 log_10_ decrease in viral titer within *O. porcinus porcinus* ticks compared to the parental virus (34). CD2v, the protein responsible for viral hemadsorption (HAD) in ASFV strains displaying the HAD phenotype, has also been demonstrated to possess an important function in virus-tick interaction. Restoration of the HAD phenotype to the non-hemadsorbing NH/P68 strain carrying a CD2v gene interrupted by frameshift mutations results in an ~1,000-fold increase in viral titer within *O. erraticus* ticks after feeding on infectious whole blood, most likely due to effects on virus uptake and replication in the tick midgut epithelium (35).

Studies of ASFV infection and replication in soft ticks show that ASFV infection takes 15–21 days to reach the midgut epithelium where viral replication is initiated, with peak virus titers achieved by 28 days post-infection (25). Restricted replication within midgut epithelial cells reduces the infectivity of the Malawi Li 20/1 strain for soft ticks orally exposed to the virus (36). For successful transmission, ASFV replication in the coxal and salivary glands is required, which is usually achieved by 48 days post-infection (25).

**Figure 2:**
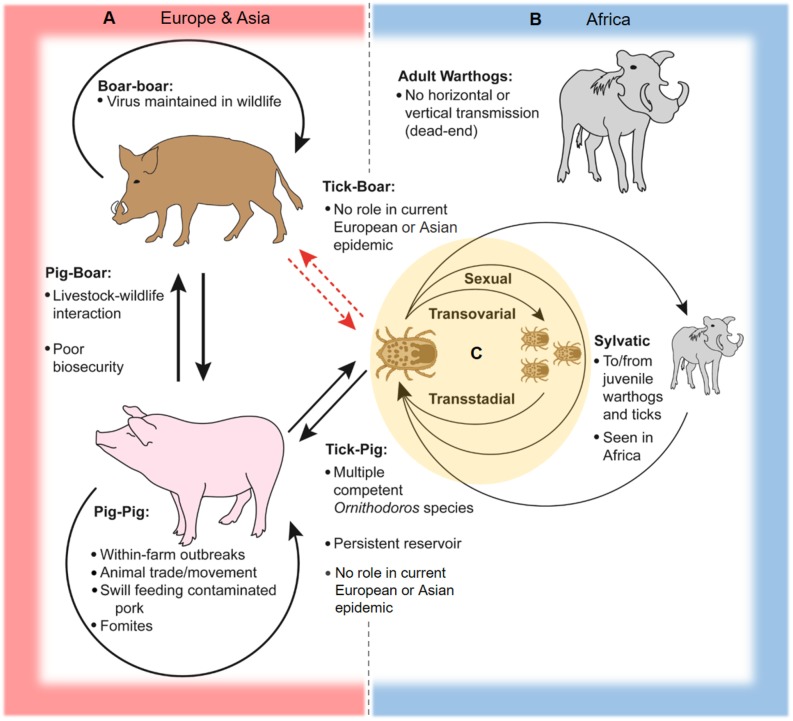
Schematic of ASFV transmission cycles. In Europe, Asia, and Africa, ASFV is readily transmissible between domestic pigs through direct contact and contaminated pork products and fomites. **(A)** In Europe and Asia, two-way transmission between pigs and boars can occur at the livestock-wildlife interface, especially where poor farm biosecurity exists. Transmission between wild boar is capable of maintaining and spreading the virus across large geographic areas. ASFV can be transmitted between soft ticks of the *Ornithodoros erraticus* complex and domestic swine, and soft ticks can serve as persistent reservoirs for the virus as seen in the Iberian Peninsula. There is little evidence to support transmission between soft ticks and Eurasian wild boar and domestic pigs in contemporary European and Asian epidemics. **(B)** The sylvatic cycle in Africa involves virus transmission between juvenile warthogs (*Phacochoerus africanus*) and soft ticks of the *Ornithodoros moubata* complex. Infected ticks transmit ASFV to juvenile warthogs when taking a blood meal, and uninfected ticks are infected after feeding on viremic juvenile warthogs, while adult warthogs typically do not maintain high levels of viremia and are dead-end hosts. **(C)** Within soft ticks of the *O. moubata* and *O. erraticus* complexes, virus is transmitted via sexual and transovarial routes and can be maintained across multiple life stages.

Within the tick life cycle, ASFV can be transmitted sexually from infected male to female (17, 32), transovarially from infected female to offspring (15, 27, 37), and maintained transstadially through the various life stages [(28, 29, 38, 39); see Figure 2]. An increase in mortality rates in ASFV-infected ticks has been reported during the first three ovipositions (18, 32). The number of infected ticks observed under field conditions is typically lower than infection rates observed after experimental infections (18, 40).

### ASFV Sylvatic Cycle

In Africa, ASFV is mainly maintained in a sylvatic cycle between *Ornithodoros* soft ticks and warthogs (*Phacochoerus africanus*); warthogs become viremic but do not develop clinical disease after ASFV infection (3, 19, 22, 41). The sylvatic cycle has been documented primarily for countries in southern and eastern Africa (3). Juvenile warthogs dwelling in burrows are infected by soft ticks carrying the virus, and transmission to naive ticks occurs when the ticks take a blood meal from viremic young warthogs [(14, 41); Figure 2]. ASFV warthog blood titers of at least 10^3^ HAD_50_/mL are required to infect feeding ticks, which is typically only achieved in young warthogs compared to adults which rarely have ASFV titers above 10^2^ HAD_50_/mL (19, 41). Other wild suids in Africa such as bush pigs (*Potamochoerus larvatus*) can also become infected and transmit ASFV, but are generally considered to play a minor role compared to warthogs in the sylvatic cycle since their behaviors are less conducive for interactions with soft ticks; only one incidence of infection in a giant forest hog (*Hylochoerus meinertzhageni*) has been reported (3, 19, 22, 42, 43).

### Tick-Pig (*Sus scrofa domesticus* and *Sus scrofa*) Transmission

*Ornithodoros* soft ticks including species of *O. moubata* complex in Africa and *O. erraticus* in Europe are capable of transmitting ASFV to domestic swine (*Sus scrofa domesticus*), and can become infected after feeding on viremic animals [(22, 25, 29, 44); Figure 2]. In Africa and Madagascar, infected ticks of the *O. moubata* complex have been isolated from pig sties and farms in locations affected by ASF outbreaks, including sites where little or no contact between wild and domestic swine occurs, suggesting an important role for soft ticks in disease maintenance in these areas (20, 45–47). A similar pattern was also observed in the Iberian Peninsula, where *O. erraticus* ticks were associated with the persistence of ASFV (7, 21–23, 40).

The genotype II Georgia 2007/1 strain responsible for the contemporary European epidemic has been experimentally demonstrated to replicate efficiently in live *O. erraticus* ticks (48). However, it is unlikely that a soft tick cycle plays a significant role in the ongoing outbreak in Europe and most likely also Asia, as soft ticks are largely absent in Central Europe and the Baltic nations, and most of the soft tick species in Eastern Europe and the Caucasus region do not infest domestic and wild swine (49). A study investigating potential contact between wild boar and soft ticks in Germany via serum screening for antibodies against *O. erraticus* in wild boar showed little evidence for feeding and infestation of wild boar by soft ticks, and limited interaction between these ASFV hosts is assumed (50).

### Domestic (*Sus scrofa domesticus*) and Wild Boar (Eurasian Wild Pig; *Sus scrofa*)

#### ASFV Infection in Domestic Swine

Infection with ASFV can produce a variety of clinical presentations ranging from chronic, subclinical, or low-level disease to hemorrhagic fever and peracute death, depending on viral strain, and host susceptibility (51). Studies of highly virulent Eurasian genotype II isolates have produced mortality rates of 100% in domestic pigs and wild boar, with disease rapidly progressing from non-specific clinical symptoms (fever, depression, anorexia, diarrhea) to death (52, 53). In contrast, the non-fatal genotype I isolates OUR T88/3 and OUR T88/4 obtained from *O. erraticus* ticks on a farm in Portugal produce no clinical disease after experimental infection of pigs (44). Genotype I ASFV strain NH/P68, isolated from a chronically-infected pig, is another example of a naturally occurring, non-fatal ASFV strain (54). However, attenuated ASFV strains including OUR T88/3 and NH/P68 can cause chronic infection in some pigs and have been associated with chronic lesions affecting the skin and joints (54–58). Swine populations displaying increased resistance to clinical ASF have been previously described; however, offspring from these pigs reared in quarantine facilities showed no difference in survival rates compared to non-selected, susceptible animals after virulent ASFV challenge, suggesting resistance is not directly heritable (59). Clinical outcomes of ASFV infection are therefore influenced by a variety of host, virus, and epidemiological factors.

#### Domestic Pig-Wild Boar Transmission

Domestic pigs readily transmit ASFV to other susceptible swine, and outbreaks of virulent strains display high levels of morbidity and mortality (22). Direct contact with infected pigs effectively spreads disease to other wild and domestic pigs (22, 60); however, varying levels of transmission efficiency have been observed for high-, moderate-, and low-virulence strains, likely due to differences in levels of viremia and virus shedding (44, 60–63). Blood, body fluids, feces, and carcasses of infected pigs serve as indirect routes of infection (60). Animals which recover from infection with low or moderate virulent strains can become subclinical carriers potentially capable of spreading the virus to other pigs (60, 61, 64, 65). The illegal movement of infected pigs by producers or pork products has played a significant role in outbreaks of ASF in Africa, Europe, and Asia (9, 11, 66).

Eurasian wild boar are highly susceptible to the virulent ASFV genotype II isolates circulating in Europe (52, 53), and contact between infected wild boar and domestic pigs has been a significant contributing factor to the spread of ASFV in Eastern Europe, the Caucasus and the Russian Federation, where small-scale backyard pig farms with poor biosecurity are common (9). ASFV has been detected in wild boar throughout Eastern and Central Europe, and as far west as Belgium (1, 10). The existence of a geographically widespread wild pig population in which ASFV can circulate poses a significant challenge to disease control and eradication efforts.

#### Other Modes of Transmission

ASFV is stable under extreme environmental conditions, allowing it to be easily spread and transmitted. Modes of transmission other than direct contact with infected swine, tissues, carcasses or bites from infected soft ticks, include importation of infected pork products and contamination of fomites such as feed, equipment, vehicles, and clothing (22). ASFV can remain viable in a variety of animal feed ingredients under a range of environmental conditions, including those characteristic of trans-Atlantic shipping routes (67, 68), and efficient disease transmission through ASFV-contaminated liquids and plant-based animal feeds has been experimentally demonstrated (69). The movement of contaminated pork products and swill-feeding of domestic swine have been important epidemiological factors in ASFV outbreaks in the Caucasus and Russian Federation as well as the emergence of the disease in China (9, 11).

## Molecular Properties of ASFV

ASFV has a large double-stranded DNA genome ranging from 170 to 190 kilobase pairs (kbp) that encodes more than 150 open reading frames (ORFs), depending on viral strain; it is the only known DNA arbovirus (70, 71). The observed significant differences in genome size are primarily due to gain or loss of gene copies belonging to the multigene families (MGFs) and variation within the number of tandem repeats in non-coding regions of the ASFV genome (70, 71). Mass spectrometry has identified 68 virion-associated structural proteins from purified virions of strain BA71V and up to 94 virion-associated polypeptides were detected in virions from 3 different mammalian cell lines infected with a recombinant OURT 88/3 strain; the precise function of a significant proportion of the structural and non-structural ASF viral proteins is unknown (72, 73). The virion is ~200 nm in diameter and possesses a multi-layered structure consisting of the nucleoid, core shell, inner envelope, capsid, and a host-derived outer envelope (74). The p72 major capsid protein and four minor capsid proteins, M1249L, p17, p49, and H240R, make up the viral capsid (75).

Genotyping of ASFV has historically been based on the nucleotide sequence of a 478 bp variable region in the C-terminus of the viral p72 gene (76), though other viral genes have also been used to further characterize ASFV strains (77–79). Currently, there are 24 genotypes based on the major capsid protein p72, and 8 serotypes based on the viral hemagglutinin CD2-like protein (CD2v) and C-type lectin (80–83). All of the 24 ASFV genotypes have been identified in Africa (3). Strain virulence cannot be accurately predicted by p72 genotype (80). The first emergence of ASFV outside of Africa consisted of genotype I viruses, which are predominantly described in West Africa (22). Genotype II ASFV was introduced into the Caucasus in 2007, most likely from East Africa, and remains the current ASFV genotype circulating throughout Europe, the Russian Federation and Southeast Asia (22, 84).

**Figure 3:**
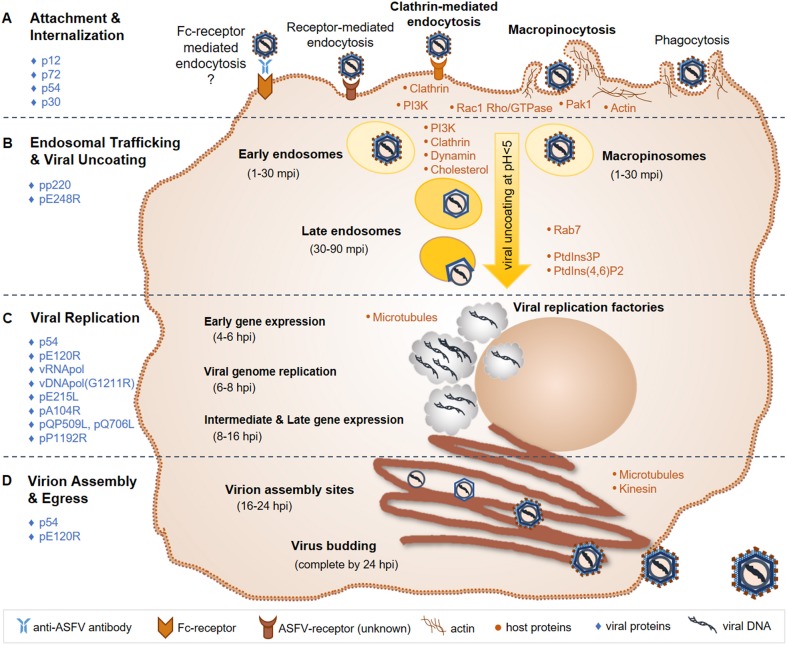
ASFV replication cycle. **(A)** ASFV entry is primarily mediated through an unknown receptor and/or macropinocytosis; Fc-receptor mediated entry and phagocytosis have also been suggested entry mechanisms. **(B)** The virus is then trafficked through early endosomes or macropinosomes to late endosomes, where viral uncoating takes place via endosomal acidification. **(C)** Viral replication takes place in the cytoplasm in viral factories, with brief replication events occurring in the nucleus. Gene expression occurs temporally, first with early genes to produce replication proteins, followed by intermediate and late genes that produce structural proteins that are assembled into the virion. **(D)** Virions are assembled and bud from the infected cell within 24 hpi. Known major host (orange dots) and viral (blue diamonds) factors involved in the ASFV replication cycle, which are discussed in the text, are indicated. ASFV, African swine fever virus; mpi, minutes post-infection; hpi, hours post-infection; vRNApol, viral RNA polymerase; vDNApol, viral DNA polymerase; PI3K, phosphoinositide-3-kinase; Rac1, Rac-1 Rho-GTPase; Pak1, Pak-1 kinase; PtdIns3P, phosphatidylinositol-3-phosphate; PtdIns (4, 6); P2, biphosphate PtdIns (4, 6) diphosphorus.

Details of virus-host interactions and events involved in the ASFV replication cycle have been reviewed previously (71, 85–88) and are summarized in the following sub-sections and Figure 3.

### Target Cells

In swine, ASFV preferentially replicates in cells of the monocyte/macrophage lineage (89). It can also replicate in some established cell lines although with less efficiency (87, 90). The virulent ASFV BA71 strain was adapted to replicate in Vero cells (BA71V). Vero cells derived from African green monkey kidney, and have been widely used as a model for *in vitro* ASFV infection and replication studies (91). However, adaptation in cell culture can cause genetic modifications to the virus that result in attenuation of virulence and decreased fitness in primary swine macrophage cultures, which is the case for BA71V and other cell-adapted ASFV strains (92, 93). Therefore, only virus derived from infected animal tissues or propagated in primary swine macrophage cultures usually retains the natural characteristics and phenotype of the original virus (90).

### ASFV Entry and Early Events in the Infectious Cycle

Several modes of cell entry for ASFV have been demonstrated (86, 94–96). Early studies on ASFV entry indicate receptor-dependent mechanisms including low pH and temperature-dependent events, and determined that ASFV binding to cell surfaces was saturable (97–100). Given the cell tropism of ASFV, several macrophage receptors have been implicated as playing a possible role including CD163, CD45, MHC II, and others, although no specific receptor for ASFV has yet been identified (101, 102). Earlier *in vitro* studies supported CD163 as being a significant receptor for ASFV, demonstrating that monoclonal antibodies could block infection (101). However, ASFV infection of gene-edited pigs lacking CD163 showed no difference in the course of infection or survival compared to wild type CD163-expressing pigs, indicating that other receptors or entry mechanisms are critical (103).

Fc-receptor mediated entry of ASFV into cells has also been proposed, although the results from one study indicated Fc-receptors do not mediate ASFV infection of macrophages (104). Nonetheless, several studies suggest antibody-dependent enhancement of ASFV infection characterized by early and increased viremia and accelerated disease, supporting *in vivo* Fc-receptor involvement (105). Antibody-dependent enhancement of infection occurs through entry of macrophage-tropic microorganisms facilitated by IgG antibody-antigen complexes and Fc-receptor signaling, and has been demonstrated for several viruses including porcine reproductive and respiratory virus, Dengue virus, and West Nile virus, among others (106–110). Accelerated disease has been observed in vaccinated swine compared to non-vaccinated controls following virulent ASFV challenge (111–113), and enhancement of ASFV infection was observed *in vitro* in the presence of sera from immunized animals, all of which coincide with the presence of non-neutralizing ASFV-antibodies (111, 114). Further investigations are warranted to elucidate the mechanisms involved in ASFV enhancement of infection and pathogenesis.

Other mechanisms including phagocytosis (115) and non-receptor mediated entry by macropinocytosis have also been investigated (94, 116). Macropinocytosis is the non-selective, actin-dependent uptake of molecules, and is utilized by several large DNA viruses, including poxviruses and herpesviruses (117). ASFV apparently also utilizes macropinocytosis, demonstrated by the use of chemical inhibitors, purified labeled virions, and fluorescent and transmission electron microscopy to monitor early events of ASFV infection in swine macrophages (94, 116).

The current working model for ASFV entry includes both clathrin-mediated endocytosis and macropinocytosis (86, 95, 96). Actin modulation through phosphoinositide-3-kinase (PI3K), Rac-1 Rho-GTPase and Pak-1 kinase signaling is important for ASFV internalization via macropinocytosis (85, 94, 96, 118). PI3K is essential for ASFV infection, likely playing a critical role in endosomal trafficking of ASFV (85, 94, 118). Clathrin, dynamin, and cholesterol are required for ASFV transport through endosomes in both Vero cells and swine macrophages (118–120). Following internalization into early endosomes or macropinosomes, ASFV particles are transported to late endosomes where the cellular factor Rab7 was shown to play an important role (116, 118). ASF virions can be found in early endosomes between 1 and 30 min post-infection (mpi) and in late endosomes at 30-90 mpi. Increasing acidification through endosomal trafficking plays an essential role in the uncoating of the ASFV outer envelope and capsid. A pH below 5.0 was shown to be required for virion uncoating (116). Fusion then occurs between the ASFV inner envelope and late endosomal membranes, releasing the viral core into the cytoplasm where viral factories will subsequently form and ASFV replication takes place. Host cell phosphatidylinositol-3-phosphate (PtdIns3P) and biphosphate PtdIns (4, 6) diphosphorus are important for the progression of early infection events to the start of ASFV replication (85, 118).

#### ASFV Proteins Involved in the Initial Steps of Infection

ASFV structural proteins involved in virus attachment to permissive cells include p12, p72, and p54 (121–124). ASFV p30 is an early and abundantly expressed phosphoprotein and is necessary for virus internalization (124). The pp220 polyprotein is cleaved into 4 major protein components of the viral core, all of which are required for core detachment and release (125–127). The internal envelope protein pE248R is essential for viral fusion with endosomal membranes and core release (116, 128).

### ASFV Gene Expression and Replication

Similar to other large DNA viruses such as poxviruses and herpesviruses, ASFV uses a temporal gene expression strategy (71, 85). The viral RNA polymerase recognizes and initiates the expression of early, intermediate and late genes throughout the respective stages of the ASFV replication cycle. Early gene expression occurs around 4–6 h post-infection (hpi), and produces proteins necessary for viral replication. At 6–8 hpi, ASFV replication is initiated via its own DNA polymerase encoded by gene G1211R. While ASFV replication primarily occurs in viral factories in the perinuclear region of the cytoplasm, an initial brief replication phase takes place in the cell nucleus (88). Intermediate and late gene expression then follows at 8–16 hpi producing structural proteins that are incorporated into the virion. ASFV encoded E2 ubiquitin-conjugating enzyme [E215L; (129)], histone-like protein [pA104R; (130)], RNA helicases [QP509L and Q706L; (131)], and topoisomerase II [pP1192R; (132, 133)] have all been shown to localize to viral factories as well as diffusely within the cell cytoplasm and nucleus. Localization and expression studies along with siRNA knockdown experiments indicate that these viral factors play important roles during ASFV gene expression, genome replication and packaging (134).

### Virion Assembly and Transport of Mature Virus Particles

Microtubules play an essential role in ASFV cellular transport and viral factory formation (85, 86, 135, 136). Microtubules and kinesin work together to support budding of ASF virions from the infected cell (136). ASFV p54 interacts with microtubules and is required for formation of viral factories and the recruitment of envelope precursors to virion assembly sites (137, 138). The viral capsid protein pE120R facilitates transport of mature virus particles from assembly sites to the plasma membrane, where the virus acquires its host-derived outer envelope (74, 139). Altogether, an entire ASFV infection cycle, from attachment and entry to budding of mature virus particles, is completed within 24 hpi (85).

### ASFV Gene Functions and Virulence Factors

ASFV encodes for at least 150 proteins. So far, 38 ASFV proteins are associated with known or predicted functions in nucleotide metabolism, transcription, replication and repair; more than 24 ASFV proteins are involved in virion structure and morphogenesis, and at least 8 ASFV proteins are likely involved in host cell interactions (71). However, the functions of a large number of ASFV-encoded proteins still remain unknown. ASFV encodes several gene products involved in virulence and counteracting host antiviral responses. The ASFV protein DP96R has been shown to inhibit the cGAS-STING pathway, thereby blocking IFN-β production, a key mediator between innate and adaptive immune responses (140, 141); ASFV gene product I329L has been shown to inhibit toll-like receptor 3 signaling and type I interferon induction (142). In addition, ASFV proteins CD2v and I215L block the transcription of immunoregulatory genes, and ASFV proteins DP71L, A179L, A224L, and EP153R promote cell survival (71, 85). CD2v has also been shown to bind to host adaptor protein 1 (AP-1) and localizes around viral factories, which suggests a role in subversion of cell protein trafficking to promote viral replication and packaging (143). ASFV genes of the multigene families MGF360 and MGF505/530 are also associated with counteracting antiviral host responses involving interferon-associated mechanisms (144–146), and are host range determinants (147). MGF360 genes have the most copies and are the most variable among ASFV strains (71). Naturally attenuated ASFV strains typically lack multiple copies of MGF360 and MGF505/530 genes as well as the CD2v gene (70, 71). Furthermore, it has been demonstrated that targeted deletion of certain genes within MGF360 and MGF505, or of CD2v, is capable of attenuating certain wild-type ASFV strains, but not all, indicating other ASFV virulence genes/factors are also important for the virulence of ASFV (148–151).

## Control of ASFV

Successful prevention and mitigation of ASF outbreaks is hindered by multiple factors, including the lack of an effective vaccine, the broad geographic distribution of wild and feral swine and potential arthropod vectors capable of maintaining the virus, as well as the increasingly globalized nature of animal agriculture. As a result, ASF control strategies primarily focus on early detection, restriction on livestock movement, and culling of herds affected by or potentially exposed to the virus. The development of effective countermeasures for ASF will be essential in combatting current and future epidemics, and the associated trade restrictions.

### Vaccines

Despite decades of research, a broadly protective, commercially available vaccine for ASFV remains elusive. Multiple vaccine development strategies have been employed, with varying levels of success. Inactivated whole viral antigen does not induce protective immunity (152). Subunit, vector-based, and DNA vaccines targeting specific viral proteins have produced inconsistent results, ranging from variable protection to enhancement of disease and accelerated mortality (105, 113, 153–155). Attenuated modified live virus (MLV) vaccines, derived from extensive viral passaging in cell lines or through targeted gene deletions, have been extensively investigated and can confer protection against homologous parental virus challenge (156), but generally provide little to no cross-protection against heterologous virulent strains (157). Additionally, MLV vaccines usually have a limited safety profile with modest to severe side effects causing arthritis, skin necrosis and chronic infections. Further research into the correlates of protection and basic ASFV immunology is needed to facilitate targeted, rationally-designed vaccine development (105, 157–159). A number of highly immunogenic ASFV antigens have been identified, yet the role of ASFV-specific cellular and humoral immune responses in protection from ASF is still not completely clear. Results regarding the role of ASFV-specific neutralizing antibodies in protection are conflicting, and high levels of non-neutralizing antibodies appear to have a detrimental effect (105, 160). For example, the presence of neutralizing antibodies does not always confer protection and in some cases immunization with ASF proteins is associated with enhanced ASFV infection and pathology, despite induction of antibodies which are neutralizing *in vitro* (105, 113, 153). Importantly, cell-mediated immunity, including induction of CD8+ T-cells and natural killer cells, appears to play an important role in protection against ASF (54, 161, 162), since pigs exposed to the low-virulence OUR/T88/3 strain and subsequently depleted of CD8+ lymphocytes were no longer protected from challenge with the virulent OUR/T88/1 isolate.

Basic research to elucidate ASFV gene functions and the mechanisms of ASFV replication, pathogenesis and immune responses is critically needed to facilitate rational vaccine development (105, 157–159). This research will be important for identifying protective proteins as vaccine targets and feasible delivery systems that induce both humoral and cellular immune responses which correlate with protection. Other important elements needed for successful ASFV vaccine development are a permanent cell culture system for MLV vaccine production as well as the design of companion diagnostic assays that are capable of differentiating infected from vaccinated animals (DIVA) (157, 158).

### Detection and Diagnosis

Since its discovery over 90 years ago, an array of diagnostic assays have been developed and employed for ASF. However, current methods for ASFV diagnosis often possess significant limitations such as (i) suboptimal analytical and clinical sensitivity/specificity, (ii) inadequate ability to detect early acute or chronically-infected animals, (iii) high cost, (iv) long time intervals for receiving results, and/or (v) the need for specialized equipment and high containment facilities (163). Consequently, the development of accurate, rapid, affordable, and field-deployable highly sensitive and specific diagnostic tests for ASFV remains a significant priority.

#### Detection of Virus and Viral Antigen

Methods for detection of virus and viral antigens include virus isolation and hemadsorption (HAD), fluorescent antibody testing, and antigen detection by ELISA or lateral flow tests (163, 164). A positive virus isolation or HAD test is considered definitive for ASFV; however, both assays are expensive, require primary cell cultures, and take >7 days to complete, and are therefore only performed by a few reference laboratories (163, 165, 166). Furthermore, not all ASFV isolates are hemadsorbing and some would therefore test negative in the HAD test. Direct fluorescence antibody testing and a commercial antigen-based ELISA and lateral flow tests are available, but their utility is hampered by lower sensitivity and specificity (163, 164, 167). Because of these limitations, PCR is often the best methodology for detecting virus in clinical samples.

#### Molecular Detection of ASFV DNA

Real-time quantitative PCR (qPCR) testing is the recommended method for screening and confirmatory testing during active infection, due to high sensitivity, specificity, and sample throughput (163, 164). Both, conventional and qPCR formats targeting conserved regions of the viral p72 gene and capable of detecting multiple genotypes have been developed and validated, though real-time qPCR assays are considered preferable (164, 168–171). The two OIE-recommended qPCR assays use TaqMan or Universal Probe Library (UPL) probes, the latter of which provides greater sensitivity for animals with low level viremia (164, 169, 171). The two qPCR assays recommended by the OIE possess significantly greater sensitivity than commercially-available antigen detection ELISAs, for both experimental and field isolates, and can be useful for detecting ASFV in poorly preserved or degraded samples where virus isolation and direct antigen detection may not be viable (164, 166, 167). In addition to its use as a diagnostic method in swine, conventional and qPCR formats can be used to detect ASFV in *Ornithodoros* ticks, and are used in combination with sequencing to genotype viral isolates (40, 76, 172, 173).

Traditional real-time qPCR testing typically needs high-throughput thermocyclers and associated laboratory equipment such as automated RNA/DNA extraction instruments which are costly, difficult to transport, and require reliable access to electricity, as well as the use of reagents that must be kept cold (166, 174). Consequently, these assays are generally restricted to laboratory settings. Several challenges are associated with performing PCR in the field beyond the need for a portable, battery-operated thermocycler, which include performing nucleic acid extractions without a centrifuge/electricity, protecting samples against cross-contamination, and maintaining a cold chain for materials that may require refrigeration. Additionally, available portable thermocyclers for qPCR assays are low-throughput because they can only run a limited number of samples at one time. Selection of the proper thermostable PCR reagents and diluents can overcome some of the above mentioned issues (174).

Progress toward field-deployable molecular diagnostics for ASFV has involved research into novel DNA amplification and detection strategies, as well as the usage of portable equipment which can be run independent of electricity (e.g., GeneReach Pockit^TM^ or Biomeme Franklin^TM^), and lyophilized reagents which are stable at room temperature for several years (174). In addition, portable next generation sequencing (NGS) devices (e.g., Nanopore MinION) can be efficiently utilized to rapidly determine the genotype of PCR positive ASFV isolates and even sequence a significant part or the entire genome for downstream phylogenetic analyses.

The use of a commercial battery-powered portable thermocycler (T-COR 4^TM^) for the detection of ASFV via a real-time PCR assay has previously been evaluated (174, 175). In one study, reduced sensitivity of the portable thermocycler for clinical samples with very high Ct values on the gold standard qPCR was observed on a RT-PCR/PCR duplex assay for Classical and African swine fever viruses (175). In our hands, portable thermocyclers (e.g., GeneReach Pockit^TM^ or Biomeme Franklin^TM^) show comparable clinical and analytical sensitivity and specificity using ASF positive and negative experimental and field samples when compared to a laboratory thermocycler (Bio-Rad CFX 96). Rapid detection of ASFV is key to activate respective control measures. To address this need and provide near immediate detection of ASFV infected swine at the farm, fair, the sale barn, or the slaughter house, our group has developed a point of need (PON) molecular detection method using the USDA-approved qPCR ASFV assay for the detection of the ASFV p72 gene (170). The POCKIT^TM^ Nucleic Acid Analyzer (GeneReach USA) is a portable PCR device, which uses insulated isothermal polymerase chain reaction (iiPCR) technology and reports out plus/minus detection of the gene target for up to 8 samples within 1 h. DNA preparation is performed on the portable Taco^TM^Mini (GeneReach, USA) automatic nucleic acid extraction system using the GeneReach total NA extraction kit using a magnetic bead extraction protocol (113). EDTA whole blood was collected from swine experimentally infected with genotype II ASFV at various time points post-infection and the ASFV p72-specific qPCR was run side-by-side on the laboratory thermocycler BioRad CFX 96 and on the POCKIT^TM^ portable iiPCR device. The results from this side-by-side analysis demonstrated similar sensitivity and specificity of the laboratory and portable PCR devices for the detection of ASFV p72 in blood samples.

Isothermal amplification strategies are performed at a single temperature, thereby avoiding the need for thermal cycling of traditional laboratory thermocycler. Early research into a linear isothermal amplification assay for ASF by combining an oligonucleotide with an overlapping probe and the cleavase enzyme (Invader®) showed high specificity but poor sensitivity relative to other molecular diagnostic techniques (176). A study of loop-mediated isothermal amplification (LAMP) targeting the viral topoisomerase II gene (P1192R) showed good sensitivity near that of the OIE TaqMan real-time PCR assay and demonstrated the potential feasibility of a lateral flow device for detecting LAMP amplicons (177). Subsequent comparison of LAMP and TaqMan-based qPCR showed comparable sensitivity, depending on the cutoff value set for a positive LAMP reaction; the variability in reaction time to positivity for different samples on LAMP assays and its poor correlation with ASFV DNA levels as determined by qPCR Ct highlights the difficulty in establishing robust diagnostic parameters for LAMP assays (166). Two studies using the recombinase polymerase amplification (RPA) technique targeting the ASFV p72 gene have shown high sensitivity in this very rapid assay format that produces results in under 10 min; importantly, robust sensitivity could be maintained when the ASFV RPA assay was combined with a convenient lateral flow dipstick to detect ASFV amplicons (178, 179). Further validation of isothermal amplification assays for ASFV is needed to better understand the reliability and utility of these techniques as ASF diagnostic methods.

#### Detection of ASFV Antibodies

Duet to the lack of an available vaccine, ASFV-specific antibodies are always the result of current or prior ASFV infection (or are maternally-derived). ASFV-specific antibodies in convalescent animals can persist for months or years (163, 164). Several immunogenic proteins of ASFV have been previously identified, including both structural and non-structural proteins (55, 180–185). A variety of serological tests for the detection of ASFV-specific antibodies have been developed using multiple formats including ELISAs, immunoblots, indirect fluorescent antibody tests (IFATs), indirect immunoperoxidase tests (IPTs), and lateral flow tests (LFTs); several of these tests are recommended by the OIE for disease surveillance and for determining freedom from ASFV infection prior to animal movement (163, 164, 182, 184–190). The above-mentioned serological assays are limited by a low sensitivity in detecting ASFV-infected animals with early-stage infections (<7 days post-infection) or swine which are infected with highly virulent strains that produce peracute ASF disease and death before the induction of ASFV-specific antibodies can occur (163, 164).

ELISA is the most commonly used method for high throughput ASF serological testing, with multiple commercial and in-house formats validated as fit-for-purpose by the OIE (164). Soluble antigens from the cytoplasmic fraction of ASFV-infected Vero cells are more sensitive than semi-purified viral p72 at detecting early antibody responses and can be used in an indirect ELISA format that is well-established but requires BSL-3 biocontainment facilities capable of handling live ASFV (164, 188). Utilization of recombinant ASFV proteins as ELISA antigens circumvents the need for virus propagation in BSL-3 containment and can provide comparable or even improved sensitivity and specificity, as well as better consistency, compared to the indirect ELISA using native ASFV antigens, especially with poorly preserved sera (184, 185, 191, 192). Variable sensitivity using sera from different geographic areas of East and West Africa have been demonstrated for recombinant ASFV protein ELISAs, depending on the ASFV protein used as target antigen (185). ELISAs have also been adapted to screen other clinical samples besides serum such as oral fluids or meat juice, which are easier or less invasive to collect than serum. A modified version of the OIE recommended indirect ELISA was able to detect ASFV-specific antibodies in oral fluids from pigs experimentally inoculated with the attenuated genotype I strain and challenged with virulent genotype II ASFV, albeit with reduced sensitivity relative to serum; this is likely due to the comparatively lower level of ASFV-specific antibodies in oral fluids (193). Indirect ELISAs using semi-purified p72 derived from genotype I ASFV isolate BA71V grown in Vero cell culture or using recombinant ASFV p30 have also shown positive reactivity with ASFV-specific antibodies present in feces from pigs infected with the attenuated Ken05/Tk1 isolate (194). Further evaluation of fecal, meat juice and oral fluid specimens collected from experimentally and field-infected pigs are needed to assess their viability and reliability as diagnostic samples for serological testing.

Confirmatory testing using an alternative serological or antigen/virus detection assay is recommended for ELISA-positive serum, especially for endemic areas, and/or poorly preserved samples (164). The IFAT is an established confirmatory test utilizing African green monkey kidney cells (Vero cells) infected with culture-adapted viral (e.g., BA71V) and a fluorophore-conjugated secondary antibody capable of detecting swine immunoglobulins (Ig). It provides a high level of specificity by allowing direct visualization of antibody reactivity with intracellular ASFV antigens in virus factories of infected cells, facilitating discernment from background noise (164, 189, 195). The ASFV IPT is conceptually similar to IFAT and has comparable sensitivity and specificity but instead uses a peroxidase-tagged secondary anti-swine Ig detection antibody, thereby avoiding the requirement for a fluorescent microscope and facilitating larger scale testing (164, 186). The IPT has been shown to possess greater sensitivity in detecting early ASFV-specific antibody responses than the OIE-recommended indirect ELISA and multiple commercial ELISAs (167). Both the IFAT and IPT are OIE-recommended confirmatory serological tests recommended for ELISA-positive samples from areas free of ASFV and for inconclusive ELISA samples from endemic areas; the IPT is considered preferable over the IFAT (164). Immunoblots (IBs) or Western blots (WBs) use soluble cytoplasmic ASFV proteins as antigens, and can be used as an alternative to the IFAT and the IPT. They are highly specific and not too difficult to interpret since the immunoreactive proteins of ASFV detected by antibodies in the IB/WB test have been well-described (164, 187). Antibodies from positive animals maintain reactivity on IBs for longer than with the OIE-recommended indirect ELISA when the test serum is stored at room temperature or 37°C; the IB can be advantageous for poorly-preserved sera samples or where reliable refrigeration is not available (196). IBs using recombinant p54, a highly immunogenic ASFV protein expressed in *Escherichia coli* or baculovirus systems, have been described, and are easier to interpret than ASFV-infected cell-based IBs, and avoid the need for ASFV antigens produced in cells (182, 191). *E. coli*-expressed p30 has also been demonstrated to be a highly sensitive and specific antigen for IBs, capable of detecting serological responses as early as 6–8 days post-infection (197). IBs/WBs based on individual ASFV proteins do not offer the multiple ASFV antigen array present in ASFV-infected cell lysate. Therefore, future IB/WB approaches for ASF serological diagnosis should include multiple recombinant ASFV antigens in order to increase specificity and sensitivity.

## Concluding Remarks

ASFV is a complex DNA arbovirus having a significant impact on the global swine industry. The lack of a safe and efficacious vaccine and the reliance on culling of herds to prevent disease spread has resulted in in significant economic losses. Therefore, improved early detection, and on-farm biosecurity measures, as well as movement control continue to be of significant priority. Further studies on ASFV gene functions, virus and cellular factors involved in ASFV replication, pathogenesis, as well as host immune responses to determine the correlates of protection, will be critical for the development of a rationally-designed, safe, efficacious, and DIVA-compatible ASFV vaccine. In addition, given the vast distribution of susceptible soft tick vectors, wild boar, and feral pigs, methods to prevent and control ASFV establishment, and spread in populations of these species are also critically important.

## Online Citations

ChinaDaily.com.cn, 9/11/2019, “Swine fever may affect pork for several years,” global.chinadaily.com.cn

FAO situation update, 02/19/2020, www.fao.org

FAO press release, 09/08/20190, “One year on, close to 5 million pigs lost to Asia's swine fever outbreak”

Global Times, 09/18/2019, “A global battle against African swine fever,” www.globaltimes.cn

OIE WAHIS African Swine Fever (ASF) Report: September 13–26, 2019; www.oie.int

OIE WAHIS Interface, Disease information, Disease Timelines, 10/23/2019, www.oie.int

OIE WAHIS Interface, Disease information, Immediate notifications and Follow-ups, 09/21/2019, www.oie.int

Reuters Health News, 09/18/2019, “Thailand culls 200 pigs amid heightened fears over African swine fever,” www.reuters.com

Vet times, 11/19/2019, “African swine fever confirmed close to Germany,” www.vettimes.co.uk

## Author Contributions

All authors contributed to the conceptualization, writing and review of the manuscript, and are accountable for the content of this work.

## Conflict of Interest

The authors declare that the research was conducted in the absence of any commercial or financial relationships that could be construed as a potential conflict of interest.
